# Group B *Streptococcus* Engages an Inhibitory Siglec through Sialic Acid Mimicry to Blunt Innate Immune and Inflammatory Responses *In Vivo*

**DOI:** 10.1371/journal.ppat.1003846

**Published:** 2014-01-02

**Authors:** Yung-Chi Chang, Joshua Olson, Federico C. Beasley, Christine Tung, Jiquan Zhang, Paul R. Crocker, Ajit Varki, Victor Nizet

**Affiliations:** 1 Glycobiology Research and Training Center, University of California, San Diego, La Jolla, California, United States of America; 2 Department of Pediatrics, University of California, San Diego, La Jolla, California, United States of America; 3 Department of Medicine, University of California, San Diego, La Jolla, California, United States of America; 4 Division of Cell Signalling and Immunology, College of Life Sciences, University of Dundee, Dundee, United Kingdom; 5 Department of Cellular and Molecular Medicine, University of California, San Diego, La Jolla, California, United States of America; 6 Skaggs School of Pharmacy and Pharmaceutical Sciences, University of California, San Diego, La Jolla, California, United States of America; 7 Rady Children's Hospital, San Diego, California, United States of America; Boston Children's Hospital, United States of America

## Abstract

Group B *Streptococcus* (GBS) is a common agent of bacterial sepsis and meningitis in newborns. The GBS surface capsule contains sialic acids (Sia) that engage Sia-binding immunoglobulin-like lectins (Siglecs) on leukocytes. Here we use mice lacking Siglec-E, an inhibitory Siglec of myelomonocytic cells, to study the significance of GBS Siglec engagement during *in vivo* infection. We found GBS bound to Siglec-E in a Sia-specific fashion to blunt NF-κB and MAPK activation. As a consequence, Siglec-E-deficient macrophages had enhanced pro-inflammatory cytokine secretion, phagocytosis and bactericidal activity against the pathogen. Following pulmonary or low-dose intravenous GBS challenge, Siglec-E KO mice produced more pro-inflammatory cytokines and exhibited reduced GBS invasion of the central nervous system. In contrast, upon high dose lethal challenges, cytokine storm in Siglec-E KO mice was associated with accelerated mortality. We conclude that GBS Sia mimicry influences host innate immune and inflammatory responses *in vivo* through engagement of an inhibitory Siglec, with the ultimate outcome of the host response varying depending upon the site, stage and magnitude of infection.

## Author Summary

The bacterium Group B *Streptococcus* (GBS) causes serious infections such as meningitis in human newborn babies. The surface of GBS is coated with a capsule made of sugar molecules. Prominent among these is sialic acid (Sia), a human-like sugar that interacts with protein receptors called Siglecs on the surface of our white blood cells. In a test tube, GBS Sia binding to human Siglecs can suppress white blood cell activation, reducing their bacterial killing abilities; however, the significance of this during actual infection was unknown. To answer this question, we studied mice for which a key white blood cell Siglec has been genetically deleted. When infected with GBS, white blood cells from the mutant mice are not shut off by the pathogen's Sia-containing sugar capsule. The white blood cells from the Siglec-deficient mice are better at killing GBS and are able to clear infection more quickly than a normal mouse. However, if the mice are given an overwhelming dose of GBS bacteria, exaggerated white blood activation can trigger shock and more rapid death. These studies show how “molecular mimicry” of sugar molecules in the host can influence a bacterial pathogen's interaction with the immune system and the outcome of infection.

## Introduction

Group B *Streptococcus* (GBS, *S. agalactiae*) is a Gram-positive encapsulated bacterium colonizing the vagina of 15–30% of healthy women. GBS is a leading cause of neonatal pneumonia, septicemia, and meningitis [1], [2], [3], and GBS colonization during pregnancy increases the incidence of preterm rupture of membranes and premature delivery [4], [5]. A decrease in the incidence of early-onset GBS disease in many developed countries has occurred following implementation of universal antenatal culture screening and use of intrapartum antibiotic prophylaxis (IAP) [6]. In contrast, the use of IAP has not had a similar impact on the incidence of late-onset disease, which now represents approximately one-third of total cases [6]. Up to 50% of late-onset GBS cases develop meningitis, which carries a very high incidence of neurocognitive sequelae – among survivors 13% had severe, 17% moderate and 18% mild disability at 5 years [7].

A systematic review of incidence data since 2000 estimated an overall incidence of GBS infection in infants less than 3 months of age of 0.53 cases per 1,000 births in the European region and 0.67 cases per 1,000 births in the Americas [8]. Less developed countries reporting no IAP use have an overall a 2.2-fold higher incidence of early-onset infection compared with those reporting any use. Overall case fatality rates are approximately 7–10% in this recent meta-analysis [8]. Of concern, the emergence of antibiotic-resistant GBS strains has been recently reported, likely a consequence of widespread administration of IAP [1], [9], [10]. Moreover, invasive GBS infections occurring in non-pregnant adult populations, especially the elderly and immune-compromised, have been documented with increasing frequency [11], [12], [13].

GBS expresses a capsular polysaccharide (CPS) that is a major virulence factor contributing to evasion of host immune defense mechanisms [14]. GBS CPS can be divided into ten serotypes (Ia, Ib, II–IX), each with unique structural and antigenic features. Nevertheless, all the GBS CPS repeating units share a critical conserved element: a terminally capped sialic acid (Sia). Sia is a nine-carbon backbone sugar present abundantly in the surface glycocalyx of all mammalian cells, and in this manner GBS decorates its surface with a common host epitope – a form of molecular mimicry. The sialylated CPS is recognized as a critical factor for GBS survival *in vivo* [15], interfering with host complement system functions to block C3b deposition and limit C5a production [16], [17].

An important facet of Sia biology is the function of Sia-binding immunoglobulin-like lectins (Siglecs), receptors that are largely expressed across the major leukocyte lineages to mediate important innate and adaptive immune functions [18], [19], [20], [21]. A major subset is the CD33-related Siglec family (CD33rSiglecs), most of which possess a cytoplasmic domain containing both a membrane-proximal immunoreceptor tyrosine-based inhibitory motif (ITIM) and a membrane-distal ITIM-like motif [18], [22]. Studies have shown that ITIMs can be tyrosine phosphorylated to recruit Src homology 2 domain-containing tyrosine phosphatases (SHP)-1 and SHP-2, and to trigger inhibitory signaling when the corresponding receptors are cross-linked with agonist monoclonal antibodies [23], [24], [25]. This suggests that the natural function of the inhibitory CD33rSiglecs is the engagement of ubiquitous endogenous Sia motifs acting as “self-associated molecular patterns” in the host to limit the baseline activation of leukocytes [26].

Through Sia mimicry, GBS has the potential to engage inhibitory CD33rSiglecs and dampen leukocyte innate immune responses, which may promote the survival of the pathogen [27]. In earlier work, we took advantage of Sia-blocking and Sia-nonblocking monoclonal antibodies against human Siglec-9 to provide *in vitro* evidence that GBS Sia-dependent engagement of Siglec-9 could blunt neutrophil oxidative burst and extracellular trap formation [28]. The significance of this phenomenon in the context of *in vivo* infection remained unproven, in large part due to rapid evolution of CD33rSiglecs in primates, such that the composition of the CD33rSiglec family varies greatly between primates and rodents. For example, while humans have eleven CD33rSiglecs, mice only have five.

Murine Siglec-E has been broadly detected on the surface of innate immune cells [25], [29], [30], [31] and has been proposed to represent the functional paralog of human Siglec-9. Very recently, Siglec-E knockout (KO) mice have been generated and confirmed to have an inhibitory role in leukocyte activation [31]. The availability of these animals thus allows us to test for the first time *in vivo* the pathogenic consequence of Sia molecular mimicry on bacterial interactions with host inhibitory Siglecs. We report that Siglec-E indeed interacts with GBS in a Sia-dependent manner, triggering SHP-1 recruitment to its intracellular domain and diminishing GBS-triggered myeloid cell innate immune and inflammatory responses. Exaggerated responses to GBS challenge in Siglec-E KO mice represented a “double-edged sword”, limiting GBS dissemination in sublethal systemic challenge, but promoting accelerated septicemia and increased mortality upon high-dose systemic challenge.

## Results

### GBS binds to murine Siglec-E in a Sia-dependent manner

**Figure 1:**
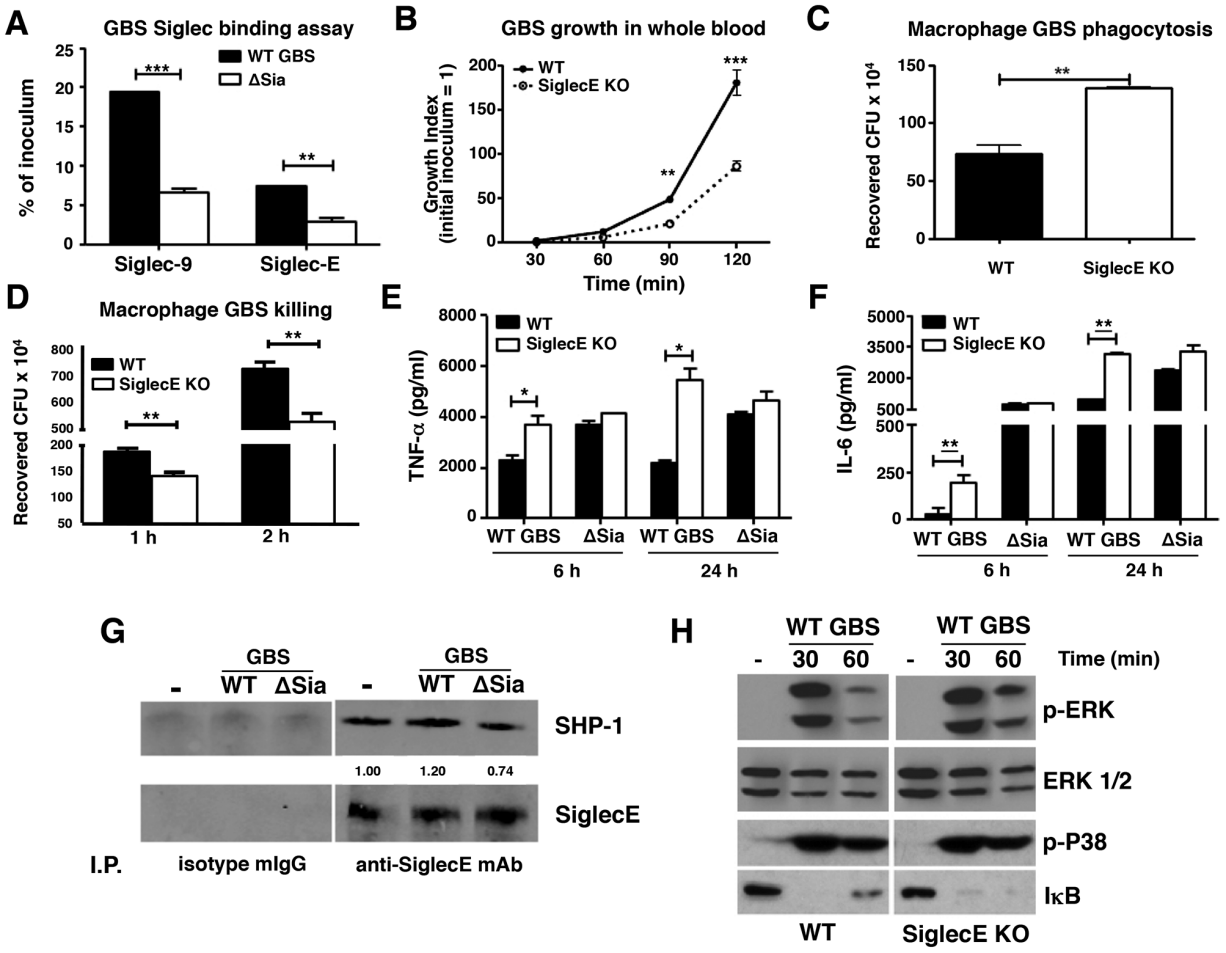
Augmented bactericidal activity in bone marrow-derived macrophages from Siglec-E KO mice. (A) Wild-type Group B *Streptococcus* (GBS WT) showed Sia-dependent binding to mSiglec-E. GBS ΔSia mutant is an isogenic mutant lacking Sia expression. Background binding from hIgG1-immobilized wells, which served as a negative control, were subtracted from data shown. (B) Whole blood from Siglec-E KO mice showed better control of GBS growth; results pooled from two independent experiments. Siglec-E KO macrophages showed greater phagocytic activity (C) and bactericidal ability (D). GBS WT but not ΔSia mutant induced higher TNF-α (E) and IL-6 (F) secretion in macrophages from Siglec-E KO animals. Differences between different groups were calculated by two-way ANOVA with Bonferroni posttest (A–B, D–F) or unpaired *t* test (C). *** *P*<0.001; ** *P*<0.01; * *P*<0.05. (G) WT GBS stimulated macrophages recruited more SHP-1 to Siglec-E than GBS ΔSia mutant. (H) Enhanced ERK activation and IκB degradation in Siglec-E KO macrophages after GBS stimulation. Representative data from two reproducible experiments are shown.

Siglec-E has been shown to bind a wide range of sialylated lipid probes that have α2,3, α2,6, or α2,8 sialyl linkages in glycan arrays [32]. We first ascertained whether Siglec-E can specifically engage GBS that expresses an α2,3-linked sialyllactosamine CPS. Fc chimeras for Siglec-E and Siglec-9 were prebound to protein A-coated plates in a high-avidity format, and FITC-labeled wild-type (WT) GBS (serotype III strain COH-1) or its isogenic Sia-deficient mutant (ΔSia) were added to the wells. The GBS WT strain but not the GBS ΔSia mutant bound to Siglec-E, the proposed functional paralog of Siglec-9, indicating that the GBS-Siglec-E interaction is indeed Sia-dependent (**Figure 1** ***A***). To confirm that Siglec-E interaction with GBS is specific for Sia, we first examined the surface charge of WT GBS and ΔSia mutant. As shown in **Figure S1*A***, the two strains bound positively charged poly-L-lysine equivalently, which indicates that GBS WT and ΔSia mutant have similar overall surface charge. In addition, we found GBS lost its Siglec-E binding ability when the side chain of CPS Sia was truncated by mild periodate treatment (**Figure S1*B***), further supporting that the GBS-Siglec-E interaction is Sia-dependent.

### Augmented innate immune responses of Siglec-E KO macrophages upon GBS infection

WT GBS engaged Siglec-9 to dampen human neutrophil activation [28]. The first question we sought to address is whether Siglec-E KO leukocytes lacking expression of this major ITIM-containing Siglec demonstrate increased bactericidal activity. WT GBS was inoculated into freshly isolated whole blood of WT or Siglec-E KO mice and bacteria colony-forming units (CFU) were enumerated at the indicated time points. As shown in **Figure 1** ***B***, growth of GBS was better controlled in Siglec-E deficient mouse blood than in WT mouse blood. Because Siglec-E has been detected in bone marrow cells, neutrophils, peritoneal macrophages and monocyte/macrophage cell lines [25], [31], we prepared bone marrow-derived macrophages (MBDMs) to study the impact of Siglec-E on the innate immune response to GBS infection. In accordance with greater restriction of GBS growth in whole blood from Siglec-E KO mice, absence of Siglec-E expression on macrophages enhanced their phagocytic (**Figure 1** ***C*** **and Figure S2**) and bactericidal (**Figure 1** ***D***) activity. In addition, WT GBS stimulated greater secretion of the pro-inflammatory cytokines TNF-α (**Figure 1** ***E***) and IL-6 (**Figure 1** ***F***) from Siglec-E deficient macrophages both at 6 h and 24 h post infection. This phenomenon was Sia-dependent, as the isogenic GBS ΔSia mutant and LPS triggered similar amounts of pro-inflammatory cytokines from the WT compared to the Siglec-E KO cells (**Figure 1** ***E*** and ***F*** **and Figure S3**).

### GBS binding to Siglec-E recruits SHP-1 and blunts activating signaling pathways

The ITIM signaling motif can antagonize kinase-dependent activation cascades by recruiting tyrosine phosphatases SHP-1 and SHP-2 [33], [34], [35]. Siglec-E is constitutively associated with a low level of SHP-1 in neutrophils, even in the absence of Siglec-E tyrosine phosphorylation [31]. We asked whether the recruitment of SHP-1 to Siglec-E can be further enhanced upon encountering the Sia-expressing pathogen GBS. WT MBDMs were incubated with or without WT GBS or the ΔSia mutant for 30 min, and Siglec-E was immunoprecipitated and probed for the co-immunoprecipitation of SHP-1. A baseline level of endogenous SHP-1 association with Siglec-E in the unstimulated MBDMs was observed, consistent with a previous report on mouse bone marrow cells [31]. The SHP-1 recruitment was slightly reduced in the ΔSia mutant-treated cells compared to that of unstimulated cells, which suggests the activation of the ΔSia mutant reduces endogenous SHP-1 association to Siglec-E in the absence of Sia-Siglec-E engagement. On other hand, enhanced SHP-1 recruitment was observed on macrophages exposed to WT GBS where Sia engagement occurs (**Figure 1** ***G***).

Bacterial infections characteristically activate pattern recognition receptors such as Toll-like receptors (TLRs) to initiate MAP kinase signaling cascades and NF-κB activation. We hypothesized that activation of MAP kinase and NF-κB in response to GBS may be increased in macrophages lacking Siglec-E-mediated inhibitory signaling. Indeed, WT GBS triggered prolonged ERK phosphorylation and IκB degradation in Siglec-E deficient macrophages than WT macrophages (**Figure 1** ***H***) but no difference was observed in p38 phosphorylation. Prolonged ERK phosphorylation and IκB degradation triggered in response to GBS challenge in the Siglec-E KO macrophages was consistent with the enhanced secretion of inflammatory cytokines (**Figure 1** **** ***E*** and ***F***), whereas the GBS ΔSia mutant triggered similar levels of ERK phosphorylation and IκB degradation (**Figure S4**). We also examined the expression of suppressor of cytokine signaling (SOCS)-1 and SOCS-3 on the WT and Siglec-E KO macrophages after GBS or LPS stimulation to exclude a potential general signaling termination defect in the Siglec-E KO macrophages. Similar SOCS-1 and SOCS-3 expression was observed on WT and Siglec-E KO macrophages after LPS and GBS stimulation, indicating that Siglec-E deficiency does not result in a pervasive defect in signal initiation and termination (**Figure S5**). Our data suggest that Sia present in the GBS surface CPS engages Siglec-E and induces SHP-1 recruitment to diminish MAP kinases and NF-κB activation. This pattern of pathogen subversion of host responses by engagement of the inhibitory Siglec was not observed in the Siglec-E KO macrophages nor in WT macrophages stimulated with the GBS ΔSia mutant.

### Skewed cytokine responses in Siglec-E KO mice challenged in a GBS intranasal pneumonia model

**Figure 2:**
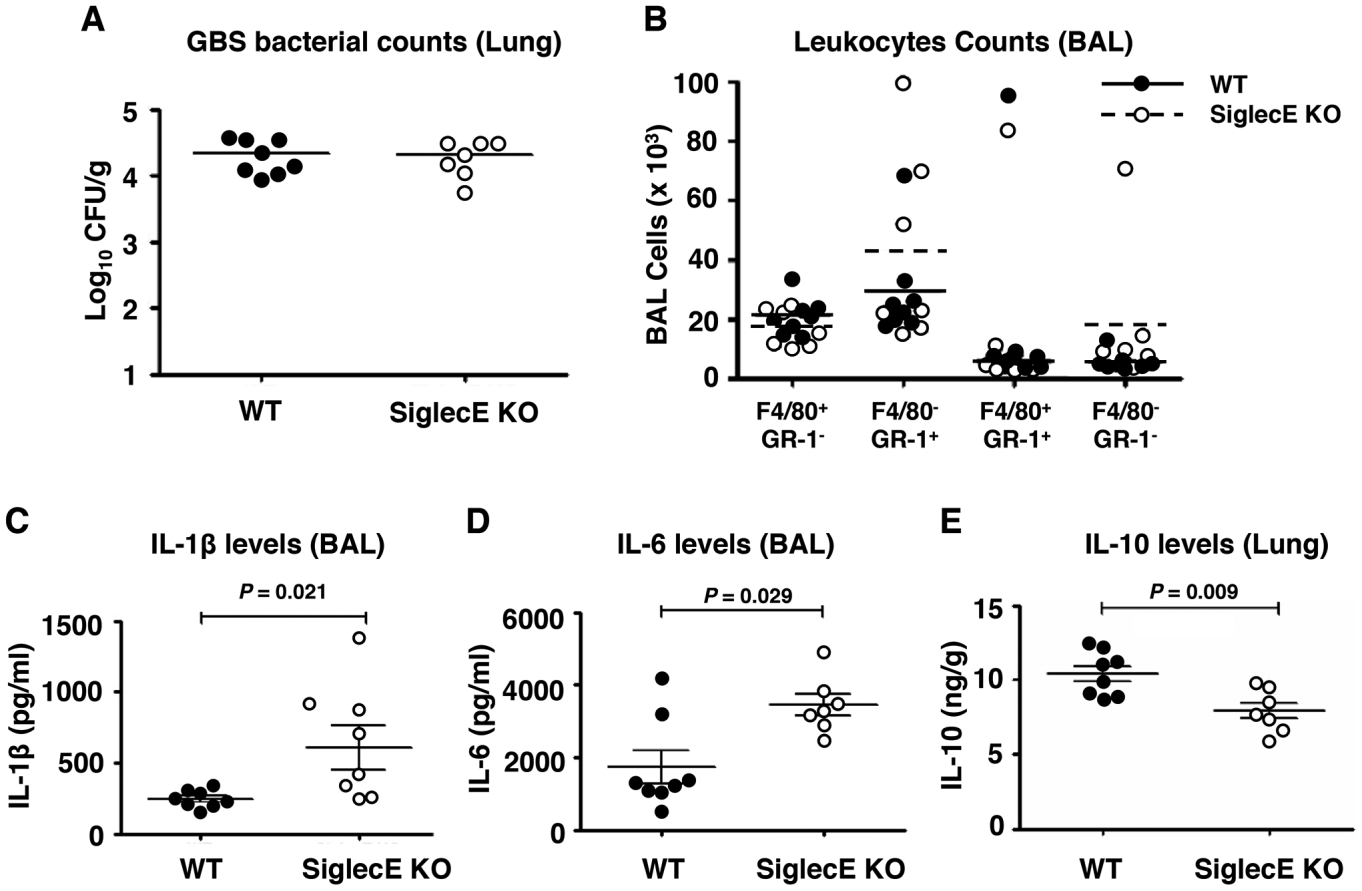
Skewed cytokine responses in Siglec-E KO mice challenged in a GBS intranasal pneumonia model. Mice were infected intranasally with 5×10^7^ CFU WT GBS and cytokine levels in cell-free BAL fluid or lung homogenates collected 6 h post infection. (A) Bacterial load in lung tissue was calculated by dilution plating for CFU enumeration. (B) Cells from BAL were counted and stained with mAb to F4/80 and Gr-1 to quantitate infiltrating cell populations. IL-1β (C) and IL-6 (D) in the BAL and IL-10 in lung homogenates (E) were examined by ELISA analysis. Data shown are means ± SEM and each circle denotes 1 mouse (n = 8 for WT and n = 7 for mSiglec-E KO mice). The difference between different groups was calculated by Mann-Whitney test.

In an earlier study, we showed that removal of the tonic inhibitory signals from Siglecs by cleaving their endogenous *cis*-ligands with pneumococcal sialidase provokes increased leukocyte inflammatory responses [36]. Since the loss of the principal inhibitory Siglec (Siglec-E) on murine macrophages increased their bacterial killing, ERK and NF-κB signaling, and inflammatory cytokine release *in vitro* (**Figure 1**), we next examined whether intranasal GBS challenge triggered exaggerated cytokine responses *in vivo*. At an early time point (6 h post-infection), no significant differences in bacteria load in lung tissues (**Figure 2** ***A***) nor infiltrated immune cells from the bronchoalveolar lavage (BAL) (**Figure 2** ***B***) were observed. However, significantly higher levels of IL-1β (**Figure 2** ***C***) and IL-6 (**Figure 2** ***D***) were found in the BAL fluid from GBS-infected Siglec-E KO mice. Moreover, a significant decrease in secretion of the anti-inflammatory cytokine IL-10 was observed in the lung tissue of Siglec-E KO mice after GBS challenge (**Figure 2** ***E***), consistent with a previous report that presence of Siglec-9 on cultured THP-1 macrophages resulted in reduced TNF-α mRNA expression accompanied with increased IL-10 mRNA levels [37]. The elevated inflammatory cytokine production and the decreased secretion of IL-10 in the Siglec-E deficient mice in response to acute localized GBS infection suggested that loss of the immunoregulatory ITIM-containing Siglec-E in leukocytes skews the host immune response toward a more inflammation prone set point.

### Absence of Siglec-E exacerbates inflammation and accelerates mortality in systemic lethal dose GBS challenge

**Figure 3:**
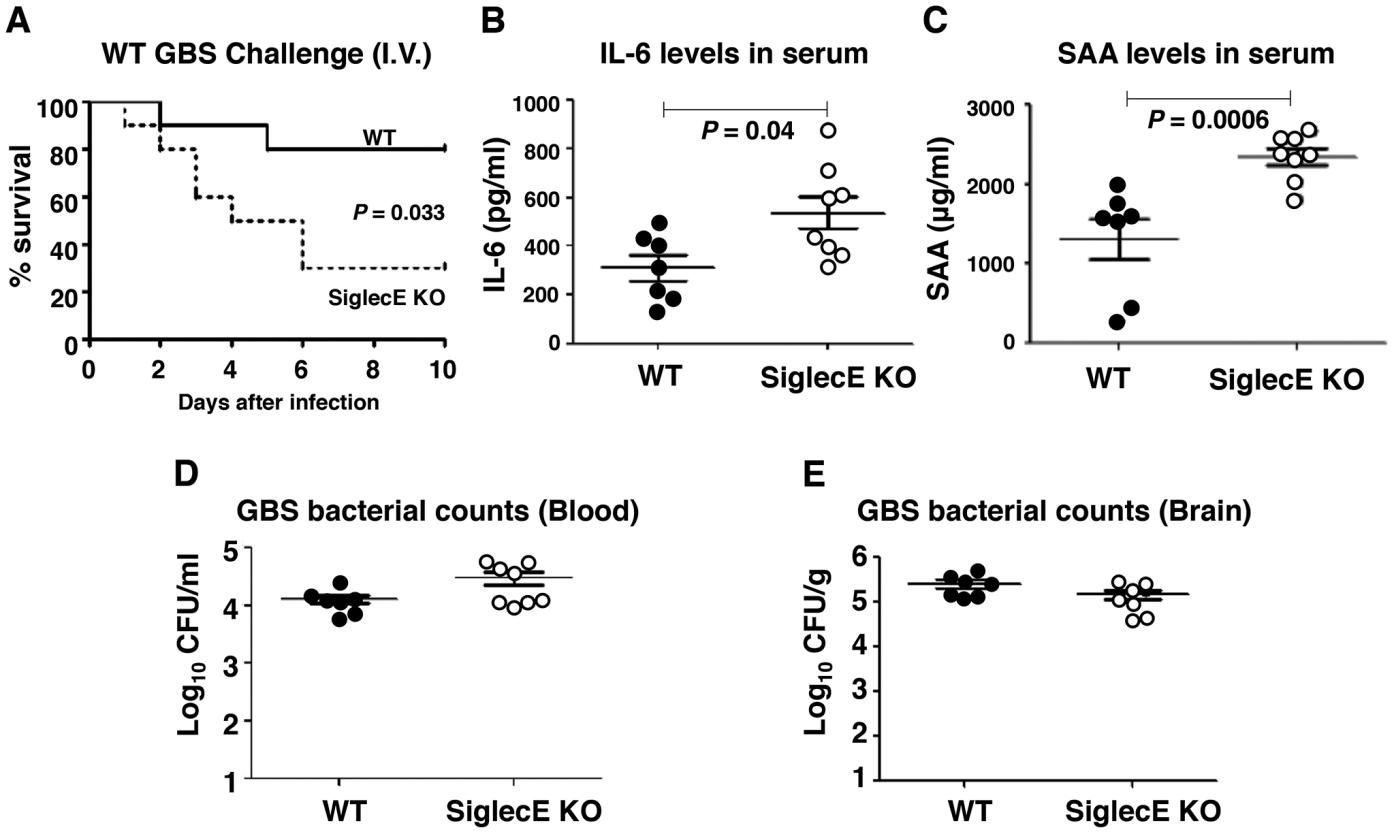
Absence of Siglec-E exacerbates inflammation and accelerates mortality in a systemic lethal dose GBS challenge. WT and Siglec-E KO mice were challenged intravenously with 3×10^8^ CFU of WT GBS. (A) Kaplan-Meier plot is shown for survival analysis (*n* = 10 for each group). Blood and brains were collected 18 h after GBS infection to measure IL-6 (B), serum amyloid A (C) and bacteria loads (D and E). Data shown are means ± SEM and each circle denotes 1 mouse (*n* = 6 for WT and *n* = 8 for Siglec-E KO mice). Differences between different groups were calculated by Mann-Whitney test (B–E).

Mice lacking Siglec-G were more susceptible to intestinal perforation-induced sepsis due to disruption of a Siglec-G/CD24 interaction, which leads to a failure in repressing host danger signaling-mediated inflammation [38]. Reduced IL-10 secretion together with excessive inflammatory cytokine production in Siglec-E KO mice upon local GBS infection let us speculate that, lacking inhibitory signals from the major Siglec expressed on macrophages, may lead to exaggerated inflammation and septicemia during high-dose systemic GBS infection. Sialylated CPS is recognized as a critical factor for GBS survival *in vivo* [15], [39], and the LD_50_ values of CPS deficient strains are up to 10^5^-fold greater than the WT strains [15], [40]; thus we are only able to model sustained infection with WT GBS and not the ΔSia mutant. When WT and Siglec-E KO mice were challenged with 3×10^8^ CFU WT GBS intravenously, 80% of WT mice survived the observation period whereas the majority of mice lacking Siglec-E died within 48 h (**Figure 3** ***A***). In parallel with reduced survival rates in Siglec-E KO animals, serum levels of IL-6 (**Figure 3** ***B***) and acute phase protein, serum amyloid A (SAA) (**Figure 3** ***C***) 18 h after infection were substantially higher in Siglec-E KO animals relative to WT controls. The increased mortality of Siglec-E KO mice was not attributable to differences in bacterial burden in the animals, since similar GBS CFU were recovered from the blood (**Figure 3** ***D***) and brains (**Figure 3** ***E***) of WT and Siglec-E KO mice. These results suggested that the accelerated fatality in Siglec-E KO mice upon high dose GBS challenge involved impaired regulation of inflammatory cytokine production.

### Reduced brain dissemination and enhanced bactericidal responses in Siglec-E deficient mice upon sublethal GBS challenge

**Figure 4:**
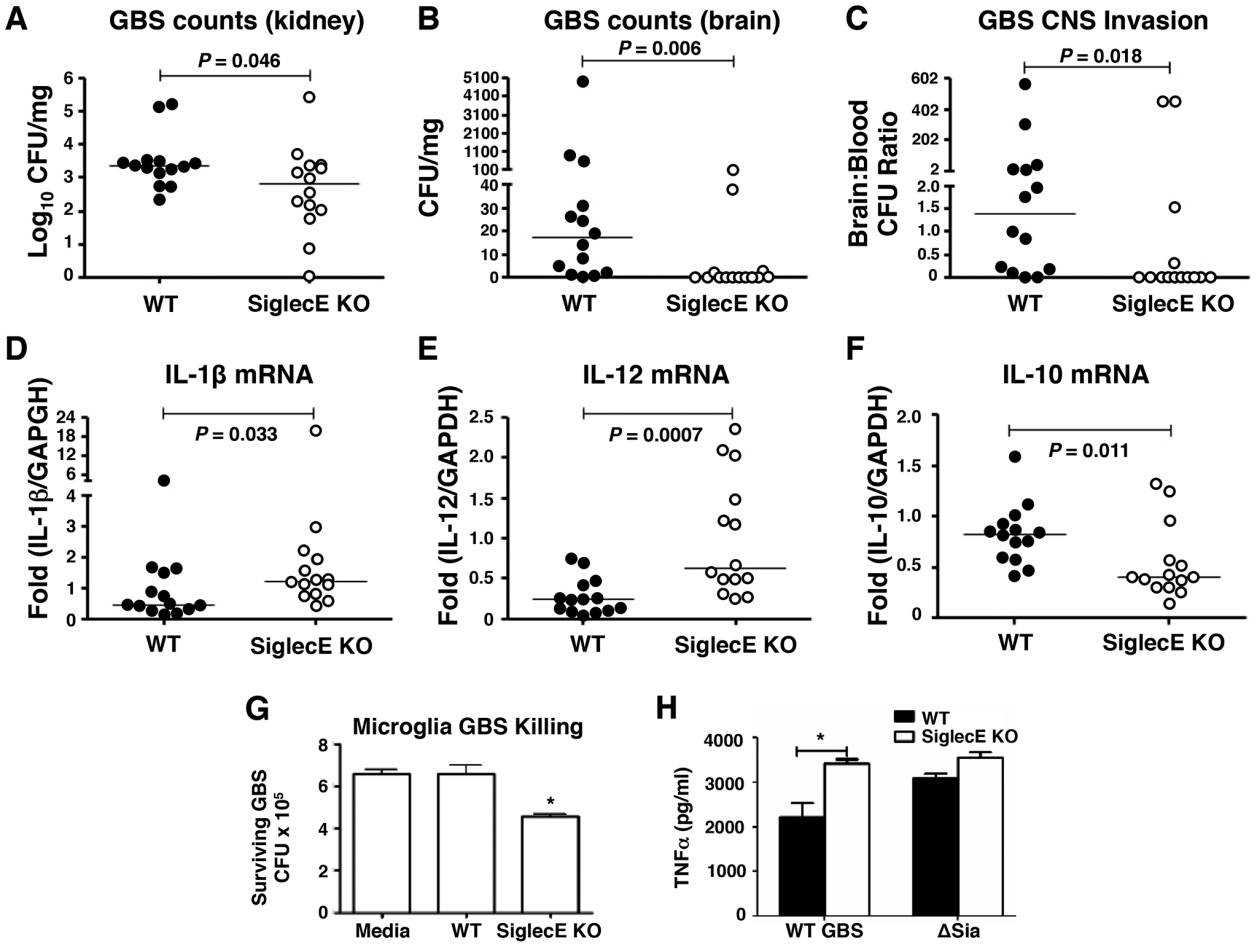
Reduced brain dissemination and enhanced bactericidal responses in Siglec-E deficient mice upon sublethal GBS challenge. Comparison of bacterial counts (expressed in CFU) recovered from the kidney (A) and brain (B) of WT mice and Siglec-E KO mice 48 h after intravenous challenge with 1×10^8^ CFU of WT GBS. (C) Brain bacterial counts were corrected for blood contamination (brain/blood ratio) using a conservative estimate of the mouse cerebral blood volume (2.5 ml per 100 g tissue). mRNA expression of IL-1β (D) and IL-12 (E) in lung and IL-10 in spleen (F) was examined by quantitative real time RT-PCR analysis. Results pooled the data from two independent experiment with final numbers of *n* = 14 for each group. Each circle denotes 1 mouse (A–F). Siglec-E KO microglia cells showed greater bactericidal ability (G) and produced higher levels of TNF-α (H) after GBS challenge. Statistical analysis was performed by Mann-Whitney test (A–F), two-way ANOVA with Bonferroni posttest (G) and one-way ANOVA with Tukey's multiple comparison test (H). *P<0.05.

Loss of Siglec-E expression was harmful for animals upon high-dose lethal GBS challenge due to excessive inflammation. However, as GBS Sia CPS is known to dampen neutrophil bactericidal activity by targeting Siglec-9 [28], we postulated that under lower-dose challenge conditions Siglec-E KO animals could benefit from loss of negative feedback on innate immunity response pathways. Consequently, WT and Siglec-E KO mice were infected with sublethal dose of GBS intravenously. GBS counts detected in the blood 4 h post-infection in each group were essentially identical, suggesting consistent establishment of systemic infection (**Figure S6*A***). None of the mice died within 48 h under sublethal GBS challenge, and infected mice were euthanized 48 h postinfection to assess bacterial dissemination to the brain and other organs. Whereas no significant overall difference in the blood GBS CFU was observed between WT and Siglec-E deficient mice, a few Siglec-E KO mice showed complete clearance of GBS in the blood (**Figure S6*B***). When compared to the WT mice, GBS-infected Siglec-E KO mice had a modest reduction in bacterial counts in the kidney (*P* = 0.046, **Figure 4** ***A***). Bacterial brain GBS CFU counts were also significantly lower in Siglec-E than in WT mice (*P* = 0.0055) (**Figure 4** ***B***), as was the ratio of CFU recovered from the brain over the bloodstream (*P* = 0.0178) (**Figure 4** ***C***).

We further measured the cytokine production in the mice after nonlethal GBS infection. Elevated serum IL-6 and SAA in the GBS-infected Siglec-E deficient mice was observed 18 h postinfection (**Figure S7*A* and *B***), although there was no difference in blood and brain bacterial loads in WT and Siglec-E deficient mice at the earlier infection stage (**Figure S7*C* and *D***). No detectable inflammatory cytokines (TNF-α, IL-1β and IL-12) were present in the serum collected at the time mice were sacrificed (48 h). The failure of cytokine detection may be due to the quick drop of systemic serum inflammatory cytokines after infection. However, quantative RT-PCR analysis revealed that the GBS-infected Siglec-E deficient mice had increased transcript levels of IL-1β and IL-12 (**Figure 4 ***D*** and ***E*****) compared to WT mice, consistent with the findings that loss of Siglec-E expression augmented inflammatory cytokine production *in vitro* and *in vivo*. Corroborating what we found in the localized intranasal pneumonia model, reduced IL-10 transcript production in Siglec-E KO mice was observed after low-dose GBS infection (**Figure 2** ***E*** and **4** ***F***). Our data show for the first time that an ITIM-containing Siglec can promote expression of the anti-inflammatory IL-10 in response to a sialylated bacterial pathogen.

### Contribution of microglia cells in the control of GBS brain dissemination

We observed reduced GBS brain CFU counts in Siglec-E KO mice after intravenous infection despite similar blood CFU counts in WT and Siglec-E KO animals. Microglial cells are the major resident macrophage population in the central nervous system parenchyma and are also part of the blood-brain barrier composition, which collectively poise them to function as a first line of defense against invading pathogens [41], [42], [43]. Since macrophages lacking Siglec-E expression exhibited greater bactericidal activity and inflammatory cytokine production, important for the control the growth and dissemination of bacteria, we tested whether microglia cells in the Siglec-E KO mice might contribute to reduced spread of GBS into the central nervous systems upon a systemic GBS infection. Microglial cells derived from WT animals showed low level expression of Siglec-E (data not shown), and loss of Siglec-E expression on microglia cells enhanced their GBS bactericidal activity (**Figure 4** ***G***). In accordance with greater inflammatory cytokine secretion in the Siglec-E deficient macrophages following WT GBS stimulation (**Figure 1** ***E*** and ***F***), WT GBS also stimulated greater TNF-α secretion the Siglec-E KO microglia cells 24 h post infection. Once again, the GBS ΔSia mutant triggered similar amounts of TNF-α secretion in WT vs. Siglec-E KO cells (**Figure 4** ***H***).

## Discussion

In the present study, we report that GBS can engage mouse Siglec-E, a functional paralog of human Siglec-9, and directly address the consequence of host responses *in vivo* in the context of presence or absence of this major inhibitory Siglec expressed on the innate immune cells during live infection with a sialylated bacterial pathogen. Loss of Siglec-E expression significantly enhanced macrophage inflammatory and bactericidal activity against GBS. The outcome and host survival was associated with the magnitude of infection and infection-induced inflammatory responses. Animals lacking Siglec-E expression appeared to benefit by the elevated inflammatory and bactericidal responses upon a low dose GBS infection. In contrast, the detrimental overwhelming inflammation accelerated mortality in the Siglec-E-deficient animals followed by a lethal dose challenge.

Leukocyte activation is controlled by a sophisticated balance between stimulatory and inhibitory signals through activating and inhibitory receptors, respectively. Bacterial infections activate pattern recognition receptors, such as TLRs, which to recognize highly conserved microbial motifs to initiate MAP kinase and NF-κB activation required for cell activation. However, the termination of such activation signals is equally critical to fine-tune the magnitude of inflammation to mitigate host cell damage, such that the overall response is dependent on all downstream signals delivered by the engaged receptors. Many inhibitory receptors contain one or more characteristic sequences (V/I*X*Y*XX*L/V) in the cytoplasmic domain classified as an ITIM. Phosphorylation of the tyrosine residue within the ITIM allows the binding of protein tyrosine phosphatases SHP-1 and/or SHP-2 or the inositol phosphatase SHIP through their SH2 domains [44], [45], [46]. When recruited to the complex, these phosphatases act to block signal transduction by dephosphorylating key proteins or lipids of a signaling cascade. Thus, regardless of the structural type of an inhibitory receptor, the inhibitory mechanism is similar. The molecular features and signaling properties of the ITIM-containing CD33rSiglecs point to a potentially important role in the limitation of excess inflammatory responses upon cell activation [21]. Reduced CD33 surface expression on human monocytes by RNA interference silencing or antibody blockade resulted in the increased secretion of IL-1β, IL-8, and TNF-α [47]. On the other hand, overexpression of WT Siglec-9, but not Siglec-9 with a mutated tyrosine into phenylalanine on the cytoplasmic ITIM motif, led to reduced TNF-α and IL-6 production upon TLR ligation [37]. In addition, ligation of Siglec-9 on monocyte-derived dendritic cells by tumor-derived Sia-containing mucins or anti-Siglec-9 antibodies suppressed IL-12 production upon LPS stimulation [48]. Similar findings can be extended to the murine Siglecs in that crosslinking of Siglec-E on LPS-treated macrophages also impaired their inflammatory cytokine production [30]. In accordance with these *in vitro* cell experiments, we found that GBS engages Siglec-E in a Sia-dependent manner to recruit SHP-1 and to influence ERK and NF-κB signaling, thereby reducing the associated inflammatory cytokine secretion and bactericidal activity (**Figure 1**). We further demonstrated that the singular loss of the major inhibitory Siglec, Siglec-E, on innate immune cells was sufficient to augment the inflammatory cytokine secretion accompanied with reduced anti-inflammatory IL-10 production during a local GBS intranasal challenge (**Figure 2**). This provides the first evidence to demonstrate that a sialylated pathogen can exploit inhibitory CD33rSiglec to negatively modulate the host inflammatory status during an *in vivo* infection.

A recent report demonstrated accelerated neutrophil and macrophage recruitment in the Siglec-E deficient lungs upon intranasal LPS administration [31]. The authors found this phenomenon was mediated through Siglec-E by suppression of CD11b outside-in signaling when Siglec-E was engaged by the sialylated CD11b ligand, fibrinogen. This Siglec-E-mediated suppression was Sia-dependent, since asiolo-fibrinogen, LPS and immuno-complex exposure did not alter the same downstream signaling observed with sialylated fibrinogen. These data examining endogenous Sias on the host fibrinogen, together with our observations regarding exogenous Sia on pathogens, emphasize the broad significance of Sia-dependent immune cell regulation through Siglec engagement.

Deficiency in inhibitory pathways results in profound immune defects characterized both by decreased activation thresholds and hyperresponsiveness phenotypes, which often lead to autoimmunity and chronic inflammation [49], [50], [51], [52]. Mice deficient in Siglec-F displayed enhanced eosinophilic inflammation [53], while animals lacking Siglec-G expression were more susceptible to intestinal perforation-induced sepsis due to failure in repressing host danger signaling-mediated inflammation [38]. In general, a fine balance must be achieved when encountering pathogens: sufficient innate immune responses must be generated in order to eliminate pathogens, but the inflammatory activation must not be too large to cause widespread host tissue damage. Upregulation of CD33rSiglecs and/or their cognate Sia ligands may represent a means for the host to attenuate and control inflammatory exacerbation by enhancing inhibitory signaling after infection. Siglec-E expression can be induced by pathogen associated molecular patterns such as ligands for TLR2, 4, 7, and 9 in a MyD88-dependent manner [30]. Reduced IL-10 secretion together with excessive inflammatory cytokine production in Siglec-E KO animals upon local GBS infection (**Figure 2**) let us evaluate whether absence of Siglec-E expression may lead to uncontrolled inflammation and tissue damages during a systemic lethal challenge. Indeed, exaggerated mortality was observed on the Siglec-E KO with higher serum IL-6 and SAA in the setting of similar bacterial burdens (**Figure 3**).

Given the role of inhibitory CD33rSiglec in controlling exaggerated inflammation, sialylated pathogens may take advantage by molecular mimicry to blunt the bactericidal functions of the immune cells in which inhibitory CD33rSiglecs are expressed through their higher-density *trans* Sia ligands. We previously reported that GBS CPS Sia mimicy allows engagement of Siglec-9 to dampen neutrophil bactericidal functions [27], [28]. Here we further demonstrate that in sublethal GBS infection, Siglec-E deficient animals exhibited reduced bacteria dissemination and may have benefited from the enhanced inflammatory responses and reduced anti-inflammatory IL-10 production. Thus GBS can trigger inhibitory signals through engaging Siglec-E to reduce overall innate immune responses in the WT animals (**Figure 4**). The established virulence function of the GBS Sia CPS is twofold, involving interference with the complement pathway [16], [17] but also downregulation of myeloid cell innate immune activities via engagement of inhibitory CD33rSiglecs.

CD33rSiglecs are expressed primarily on leukocyte subsets, and whereas some are highly restricted to a certain cell type, other CD33rSiglecs have relatively broad expression patterns. In addition, several CD33rSiglecs can be present on the same cell type in which they may perform a specific function or have functional redundancy. Since the potential contribution of each CD33rSiglec is determined primarily by its cellular expression profile, detailed comparative analyses of the specific cell types that express CD33rSiglecs are essential to clarify their unique roles [18], [54]. Here, we surprisingly found that in addition to the previously known cells that express Siglec-E, such as neutrophils and macrophages, its expression was also present on the brain microglia cells. Microglia cells are the major resident macrophage population in the CNS parenchyma and part of the blood-brain barrier (BBB) composition. The parenchymal CNS is an anti-inflammatory environment with high local concentrations of inflammation-suppressive cytokines such as IL-10 and TGF-β [55], [56]. Furthermore, the BBB is able to restrain CNS inflammation by excluding plasma proteins as well as peripheral immune cells and their associated inflammatory molecules [57]. Expression of inhibitory Siglec-11 on human microglia cells has been shown to alleviate neurocytotoxicity [58]. Therefore, Siglec-E on microglia may play a physiological role when encountering its endogenous *cis* ligands. Although microglia are believed to function as first line of defense against invading pathogens [41], [42], [43], the sialylated GBS may suppress microglial responses by engaging Siglec-E on microglia to facilitate the establishment of meningitis upon systemic GBS infection. To support this hypothesis, we found that Siglec-E KO microglia cells had greater microbicidal and cytokine responses against GBS (Fig. 4 G–H) compared to WT microglia cells.

In conclusion, our data show that GBS Sia mimicry can attenuate host innate immune responses through engagement of an inhibitory Siglec on leukocytes, with the potential outcome on the host response likely to vary dependent upon on the site, stage and magnitude of infection. In localized and early-stage infection, we propose this process can serve to diminish host macrophage innate immune functions, promoting GBS survival and virulence. In overwhelming infection, which could include fulminant neonatal sepsis after ascending infection of the placental membranes, engagement of GBS Sia by inhibitory CD33rSiglecs such as Siglec-9 could ultimately act to mitigate the dysregulated inflammatory response and cytokine storm. Similarly, microglial Siglec-E expression could reduce inflammatory responses in the privileged CNS compartment. A number of other invasive human bacterial pathogens associated with meningitis, including *Haemophilus influenzae*, *Neisseria meningitidis* and *Escherichiae coli* serotype K1, display Sia in their CPS or cell wall lipooligosaccharides with the potential to engage members of the CD33rSiglec family. Further analysis of Sia-Siglec interactions during host-pathogen encounters could provide novel targets for therapeutic intervention to modify infectious disease outcome.

## Materials and Methods

### Ethics statement

This study was carried out in strict accordance with the recommendations in the Guide for the Care and Use of Laboratory Animals of the National Institutes of Health under a protocol approved by the Institutional Animal Care and Use Committee at the University of California, San Diego (Animal Welfare Assurance Number: A3033-01). All efforts were made to minimize suffering of animals employed in this study.

### Bacteria and cell lines

A well-characterized human serotype III GBS isolate (COH-1) from a case of human neonatal sepsis was used in this study. Generation of the isogenic ΔSia mutant, containing an in-frame allelic replacement of the *neuA* gene encoding CMP-N-acetylneuraminic acid synthetase with a chloramphenicol acetyltransferase cassette, was previously described [59]. GBS were propagated in Todd-Hewitt broth, THB, (Difco, BD Diagnostics) at 37°C without shaking. For infection studies, bacteria were cultivated at 37°C to mid-exponential phase and resuspended to an OD_600_ of 0.4, followed by serial dilution and enumeration of CFU (colony-forming units) to verify each experimental inoculum.

### Siglec-Fc binding assay

Siglec-Fc and FITC-labeled GBS interactions were performed with slight modifications from our previously described method [27]. To prepare FITC-labeled GBS, early exponential phase grown GBS was pelleted, washed and then labeled with 0.1% fluorescein isothiocyanate (FITC)/PBS for 1 h at 37°C with rotation. Bacteria were extensively washed with PBS to remove trace amounts of free FITC then resuspended in PBS. 96-well plates were coated with 1 µg/well protein A in 50 mM carbonate buffer (pH 9.5) overnight at 4°C. Wells were washed and blocked with 1%BSA/PBS for 1 h at room temperature. Human Siglec 9-Fc, produced as previously described [60], or murine Siglec E-Fc [29] was immobilized to individual wells for at least 3 h at room temperature. Wells were washed three times, and 1×10^7^ FITC labeled-GBS added to each well. Plates were centrifuged at 2000 rpm for 10 min and incubated for 30 min at 37°C. The initial fluorescence intensity was verified, wells washed to remove unbound bacteria, and the residual fluorescent intensity (excitation, 488 nm; emission, 530 nm) measured using a CytoFluor^II^ fluorescent plate reader.

### Culture of mouse bone marrow-derived macrophages and *in vitro* stimulation

Murine bone marrow-derived macrophages were derived from bone marrow cells collected from femur and tibia and cultured with conditional media containing macrophage colony-stimulating factors (M-CSF) for 7 d. For phagocytosis assays, GBS (2.5×10^6^ CFU) were added to 5×10^5^ macrophages (multiplicity of infection or MOI = 5), followed by incubation for 30 min or 1 h. Cells were washed with PBS three times followed by addition of medium containing penicillin (5 µg/ml) and gentamicin (100 µg/ml) for 1 h to kill extracellular bacteria. Cells were then washed, detached, and lysed with 0.025% Triton X-100 to release intracellular bacteria. Bacterial CFU were enumerated by serial plating on the THA plates. For bacterial killing assays, 1×10^5^ GBS was added to macrophages (5×10^5^) at MOI = 0.2 for 1 or 2 h, followed by addition of 50 µl of 0.6% Triton X-100 to lyse cells. Recovered GBS were plated on THA plates for CFU enumeration. Assays were performed in triplicate and were repeated three times. To detect cytokine secretion after GBS infection, 1×10^5^ macrophages were stimulated with 10^6^ GBS for 30 min, and then penicillin and gentamicin added as above to prevent the bacterium overgrowth. The culture supernatant was collected 6 h or 24 h after GBS infection for cytokine ELISA analysis.

### Culture of microglia cells and *in vitro* stimulation

Isolation of primary microglia cells from adult animals was performed as previously described [61]. In brief, PBS-perfused brains were collected and digested in an enzymatic solution contained 116 mM NaCl, 5.4 mM KCl, 26 mM NaHCO_3_, 1 mM NaH_2_PO4, 1.5 mM CaCl_2_, 1 mM MgSO_4_, 0.5 mM EDTA, 25 mM glucose, 1 mM cysteine and 20 units/ml papain (all from Sigma) for 90 min at 37°C, followed by 0.5 mg/ml of DNase I (Roche) treatment for 5 min at room temperature. The resulted brain mixture was resuspended in 20 ml of 20% isotonic Percoll/HBSS, carefully overlaid with 20 ml HBSS and centrifuged at 200 g for 20 min with slow acceleration and no brake. The pellet containing mixed glial cells was collected, washed and cultured in the Dulbecco's modified Eagle's/F12 medium with GlutaMAX supplemented with 10% FBS, 100 units/ml penicillin, 100 µg/ml streptomycin (all from Invitrogen) and 5 ng/ml of carrier-free murine recombinant granulocyte and macrophage colony-stimulating factor (GM-CSF) (R&D Systems). The medium was changed twice a week until the cells became confluent. A nearly pure microglial cell population was collected from the mixed glial culture supernatant without any prior shaking and plated in the 48-well plates (2×10^5^ cells/well) or 96-well plates (1×10^5^ cells/well) for 3 days in medium without GM-CSF. Bacteria killing assays and cytokine secretion after GBS infection were performed as described above, using MOI of 0.2 for 2 h and MOI of 10 for 24 h, respectively.

### Cytokine detection

Concentrations of cytokines in macrophage supernatants collected over time post-infection, or cytokines in mouse BAL fluids, lung homogenates and serums from infected animals were detected by corresponding commercial ELISA kits (TNF-α and IL-6 from R'D; IL-1β, IL-10 and IL-12 from BD Biosciences; SAA (serum amyloid A) from Life Diagnostics)

### Immunoprecipitation and western blot analysis

Macrophages were lysed in lysis buffer (50 mM Tris, pH 8, 150 mM NaCl, 1% NP40) containing protease inhibitor cocktail (Roche) and phosphatase inhibitor cocktail (Santa Cruz Biotechnology). Cell lysates were then separated on a 10% SDS-PAGE and transferred to a PVDF membrane. The membrane was probed with the anti-phospho p44/42 MAPK (T202/Y204), anti-phospho p38, anti-p44/42, or anti-IκB antibodies (all from Cell Signaling Technology), and then followed by appropriate HRP-conjugated secondary Abs (Bio-Rad) and ECL reagent (Thermo Scientific). Macrophages were stimulated with COH1 WT or ΔSia mutant for 30 min at MOI = 10. Cells were then lysed in RIPA buffer with protease and phosphatase inhibitor cocktail. Siglec-E was immunoprecipitated by mouse anti-Siglec-E mAb (generated in the Crocker lab). Immunoblots were probed with Ab to SHP-1 (Santa Cruz Biotechnology) and Siglec-E (R&D systems), respectively, and then followed by appropriate HRP-conjugated secondary Abs and ECL reagent.

### Mouse infection models

All animal experiments were approved by the Committee on the Use and Care of Animals, UCSD and performed using accepted veterinary standards. For the murine intranasal challenge model, mice (Siglec-E knockout mice or C57B/6 controls, 10–12 weeks) were lightly anesthetized by intraperitoneal injection of ketamine and xylazine, and 50 µl of PBS containing 10^8^ GBS was then administered into the nostrils of the mice. The inoculum dose was confirmed by viable count after plating on THA plates. Infected animals were sacrificed 6 h post infection. Blood was collected via terminal cardiac puncture. For bronchoalveolar lavage (BAL) fluid collection, the trachea was exposed and 0.8 ml PBS (without calcium and anticoagulant) was injected twice by using an 18 guage needle connected to 1 ml syringe. Cells from BAL were counted and stained with APC-conjugated rat anti-mouse F4/80 mAb (AbD Serotec) and FITC-conjugated rat anti-mouse Gr-1 mAb (Caltag Medsystems) to analyze the infiltrated cell population after GBS infection. The left lung lobe was collected was collected for bacterium CFU enumeration and cytokine detection. For monitoring mouse survival after systemic intravenous challenge, mice (10–12 weeks) were intravenously infected with 3×10^8^ GBS and observed for 10 d. To measure serum cytokine secretion after GBS infection, blood was collected 18 h post infection for cytokine ELISA analysis, along with measurement of bacterial load in the blood and brain. For the GBS meningitis model, mice (10–12 weeks) were intravenously infected with 10^8^ GBS and, bacteria CFU in the blood was examined 4 h later to ensure mice received similar challenge doses. Then 48 h after injection, samples of blood, brain/meninges, and kidney were collected aseptically from mice after euthanasia. Bacterial counts in blood and tissue homogenates were determined by plating serial dilutions. Bacterial counts in brain and kidney were corrected for differences in organ weight. Brain bacterial counts were also corrected for blood contamination using the blood concentration and a conservative estimate of the mouse cerebral blood volume [62].

### Real time quantitative RT-PCR

RNAs were isolated from various tissues using Trizol (Life Technologies) and used as a template for cDNA preparation by iScript kit (Bio-Rad). Quantitative PCR was performed using iQ SYBR Green Supermix (Bio-Rad) according to standard protocols. Primers used were for IL-1β 5′-ACTACAGGCTCCGAGATGAAC-3′ and 5′-CCCAAGGCCACAGGTATTTT-3′; for IL-12, 5′-CGTGCTCATGGCTGGTGCAAAG-3′ and 5′-CTTCATCTGCAAGTTCTTGGGC-3′; and for GAPDH, 5′-AACTTTGGCATTGTGGAAGGGC-3′ and 5′-GGTAGGAACACGGAAGGCCATG-3′. Primers for IL-10 were obtained from QuantiTect Primer Assay (Qiagen)

### Statistical analysis

Graphpad Prism version 5 was used for statistical analysis. Statistical significance was accepted at *P*<0.05. Data shown were either pooled from two independent experiments or representative data from two independent experiments conducted with biological replicates. The significance of differences between different groups for the animal experiments was determined using the Mann-Whitney test. Unpaired *t* test, one-way ANOVA and two-way ANOVA were used for two groups, multiple groups or two parameters comparisons, respectively.

## Supporting Information

Figure S1 **WT GBS and ΔSia mutants have similar surface charge and expression of Sia is critical for the GBS-Siglec-E interaction.** (A) WT GBS (solid line) and ΔSia mutant (dashed line) were stained with FITC-labeled poly-L-lysine (Sigma) at 1 mg/ml for 20 min at room temperature and applied to FACSCalibur flow cytometer. The shaded histograms are unstained controls for each strain. (B) CFSE-labeled GBS was treated or untreated with 2 mM NaIO_4_ for 20 min at 4°C in the dark, followed by incubation with 60 mM MTSC (4-methyl-3-thiosemicarbazide) for 60 min at 37°C. The resulting treated GBS and ΔSia mutant strains were applied to Siglec-9 or Siglec-E coated plates to test their Siglec-interacting ability. Background binding from hIgG1-immobilized wells served as negative controls, and was subtracted from data shown here.
(TIF)

Figure S2 **Siglec-E deficient macrophages exhibit greater phagocytic activity against WT GBS but not GBS ΔSia mutants.** Mouse bone marrow-derived macrophages (MBDMs) were incubated with pHrodo Red (Life Technologies) labeled WT GBS or ΔSia mutant at 37°C for 30 min or 60 min at the MOI of 50 (A) or 5 (B). MBDMs were then washed three times with PBS after incubation, detached using 5 mM EDTA, and applied to FACSCalibur flow cytometer. The phagocytic activity of WT and Siglec-E deficient MBDM was reflected by the mean fluorescence intensity (MFI) of the cells, where the engulfed GBS exhibited red fluorescence once inside the phagolysosome of MBDMs. Solid and open symbols represent cells from WT and Siglec-E deficient mice, respectively. Cells infected with WT GBS are indicated in blue color, while cells infected with GBS ΔSia mutants are in green.
(TIF)

Figure S3 **Lack of Sia-Siglec-E engagement is critical for the exaggerated cytokine secretion observed in Siglec-E-deficient macrophages after WT GBS stimulation.** Mouse bone marrow-derived macrophages from WT or Siglec-E deficient mice were incubated with GBS at different MOI (from 2 to 50) or LPS at different concentrations (from 10 to 1000 ng/ml) for 24 h. Secretion of TNF-α in the culture supernatant was determined using a TNF-α ELISA kit.
(TIF)

Figure S4 **GBS ΔSia mutants induce similar level of ERK activation and IκB degradation in WT and Siglec-E deficient macrophages.** WT mouse bone marrow-derived macrophages from WT (WT) or Siglec-E deficient (E) were treated with GBS ΔSia mutants for 30 or 60 min. Cell lysates were collected at indicated times, separated on SDS-PAGE, and probed with antibodies recognizing phosphorylated form of ERK, total ERK, IκB and actin.
(TIF)

Figure S5 **Expression of suppressor of cytokine signaling -1 (SOCS-1) and SOCS-3 in WT and Siglec-E deficient macrophages can be detected after LPS and GBS stimulation.** Mouse bone marrow-derived macrophages (MBDMs) were stimulated with LPS, GBS WT and GBS ΔSia mutants for 24 h in the presence of antibiotics to prevent the overgrowth of bacteria. Cell lysates were collected, separated on SDS-PAGE, and probed with antibodies recognizing SOCS-1, SOCS-3 and actin.
(TIF)

Figure S6 **Similar blood GBS counts were observed in the systemic GBS infection model.** Comparison of bacterial counts (expressed in CFU) recovered from the blood collected from WT and Siglec-E deficient mice after intravenous challenge with 10^8^ CFU of WT GBS 4 h (A) or 48 h (B) after infection. Data shown are means and each circle denotes 1 mouse (*n* = 14 for each group).
(TIF)

Figure S7 **Enhanced proinflammatory cytokine secretion but equivalent bacterial brain dissemination in Siglec-E deficient mice upon sublethal GBS challenge at an early infection time point.** WT and Siglec-E KO mice were challenged intravenously with 10^8^ CFU of WT GBS. Blood and brains were collected 18 h after GBS infection to measure (A) serum IL-6 (A), (B) serum amyloid A (SAA), (C) blood and (D) brain bacteria loads. Data shown are means ± SEM and each circle denotes 1 mouse (*n* = 8 for each group). Differences between different groups were calculated by Mann-Whitney test.
(TIF)
