# Supplementary figures and images for: Group B Streptococcus Engages an Inhibitory Siglec through Sialic Acid Mimicry to Blunt Innate Immune and Inflammatory Responses In Vivo
Source: PLoS Pathog. 2014 Jan 2;10(1):e1003846. doi: 10.1371/journal.ppat.1003846 (PMC3879367; doi:10.1371/journal.ppat.1003846)

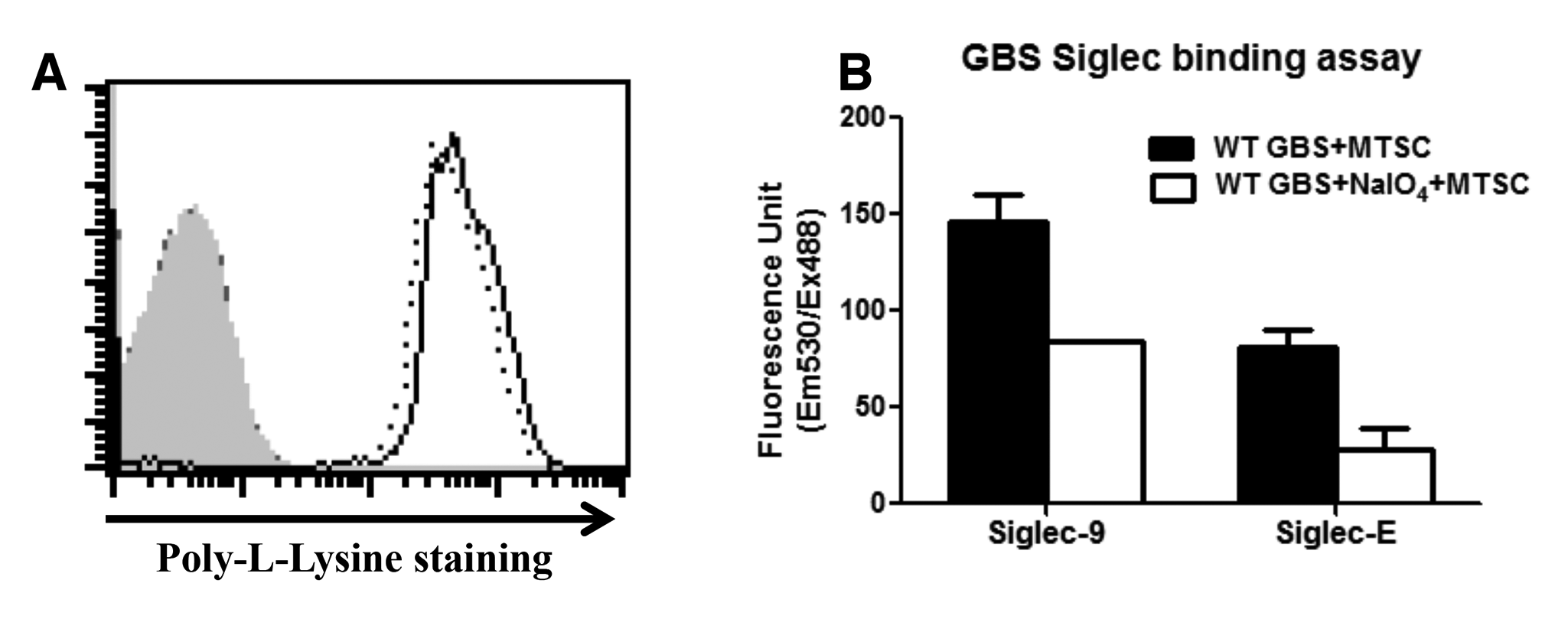

Supplement: Figure S1 — WT GBS and ΔSia mutants have similar surface charge and expression of Sia is critical for the GBS-Siglec-E interaction. (A) WT GBS (solid line) and ΔSia mutant (dashed line) were stained with FITC-labeled poly-L-lysine (Sigma) at 1 mg/ml for 20 min at room temperature and applied to FACSCalibur flow cytometer. The shaded histograms are unstained controls for each strain. (B) CFSE-labeled GBS was treated or untreated with 2 mM NaIO4 for 20 min at 4°C in the dark, followed by incubation with 60 mM MTSC (4-methyl-3-thiosemicarbazide) for 60 min at 37°C. The resulting treated GBS and ΔSia mutant strains were applied to Siglec-9 or Siglec-E coated plates to test their Siglec-interacting ability. Background binding from hIgG1-immobilized wells served as negative controls, and was subtracted from data shown here. (TIF) [file ppat.1003846.s001.tif]

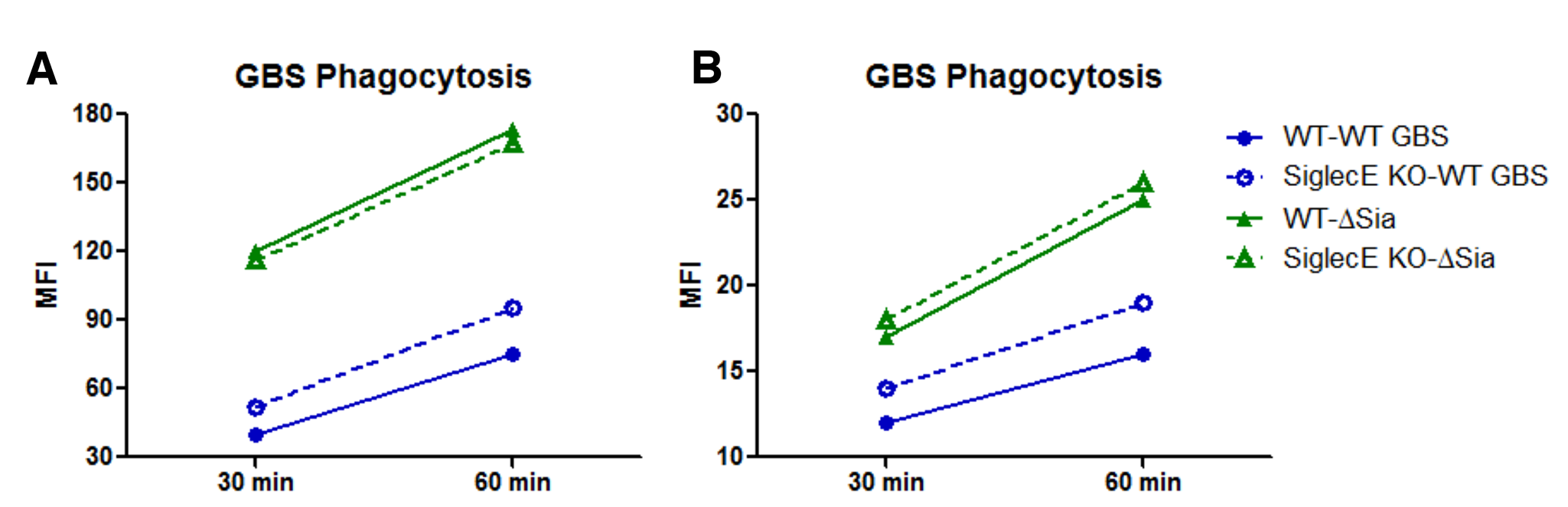

Supplement: Figure S2 — Siglec-E deficient macrophages exhibit greater phagocytic activity against WT GBS but not GBS ΔSia mutants. Mouse bone marrow-derived macrophages (MBDMs) were incubated with pHrodo Red (Life Technologies) labeled WT GBS or ΔSia mutant at 37°C for 30 min or 60 min at the MOI of 50 (A) or 5 (B). MBDMs were then washed three times with PBS after incubation, detached using 5 mM EDTA, and applied to FACSCalibur flow cytometer. The phagocytic activity of WT and Siglec-E deficient MBDM was reflected by the mean fluorescence intensity (MFI) of the cells, where the engulfed GBS exhibited red fluorescence once inside the phagolysosome of MBDMs. Solid and open symbols represent cells from WT and Siglec-E deficient mice, respectively. Cells infected with WT GBS are indicated in blue color, while cells infected with GBS ΔSia mutants are in green. (TIF) [file ppat.1003846.s002.tif]

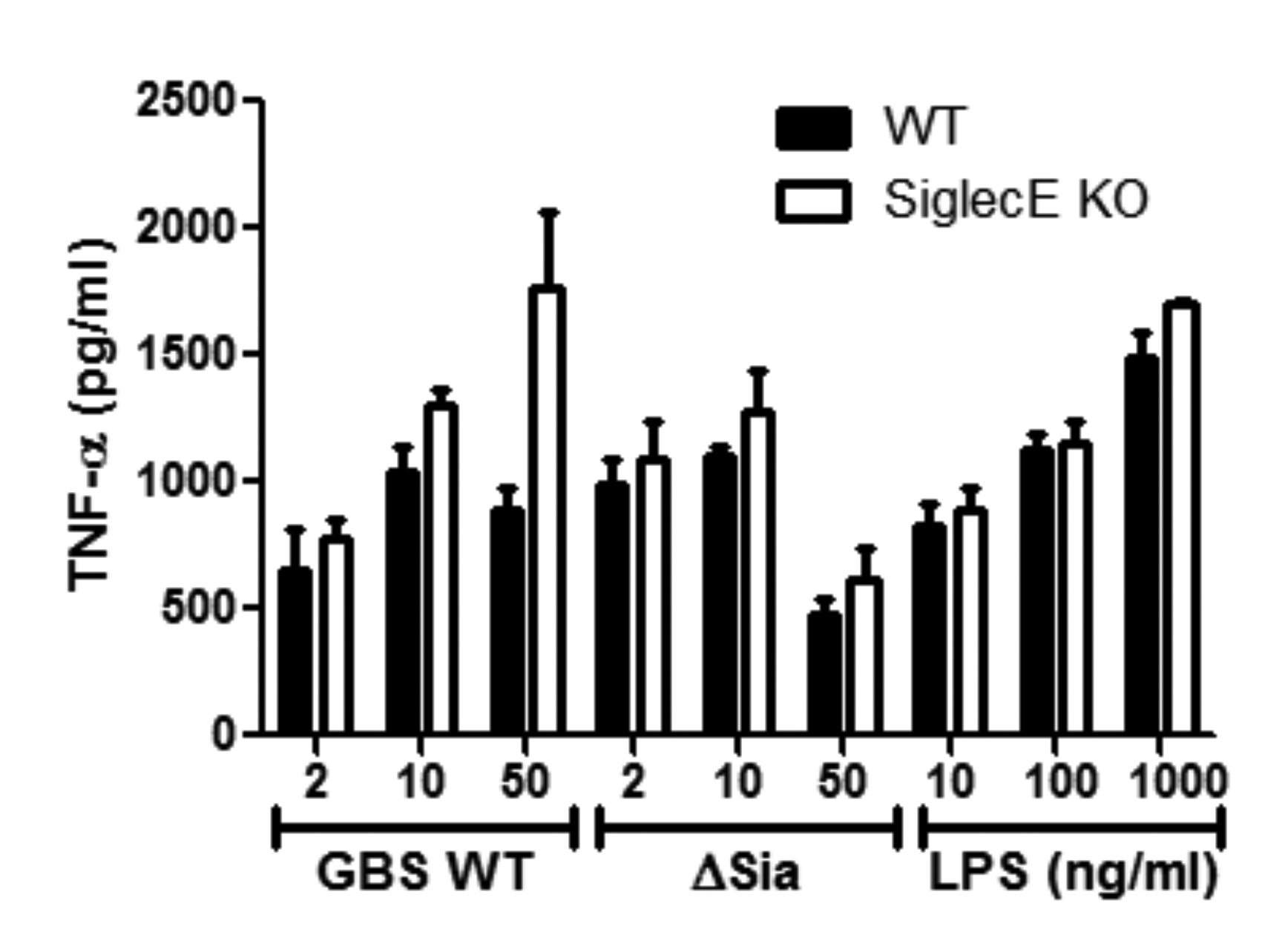

Supplement: Figure S3 — Lack of Sia-Siglec-E engagement is critical for the exaggerated cytokine secretion observed in Siglec-E-deficient macrophages after WT GBS stimulation. Mouse bone marrow-derived macrophages from WT or Siglec-E deficient mice were incubated with GBS at different MOI (from 2 to 50) or LPS at different concentrations (from 10 to 1000 ng/ml) for 24 h. Secretion of TNF-α in the culture supernatant was determined using a TNF-α ELISA kit. (TIF) [file ppat.1003846.s003.tif]

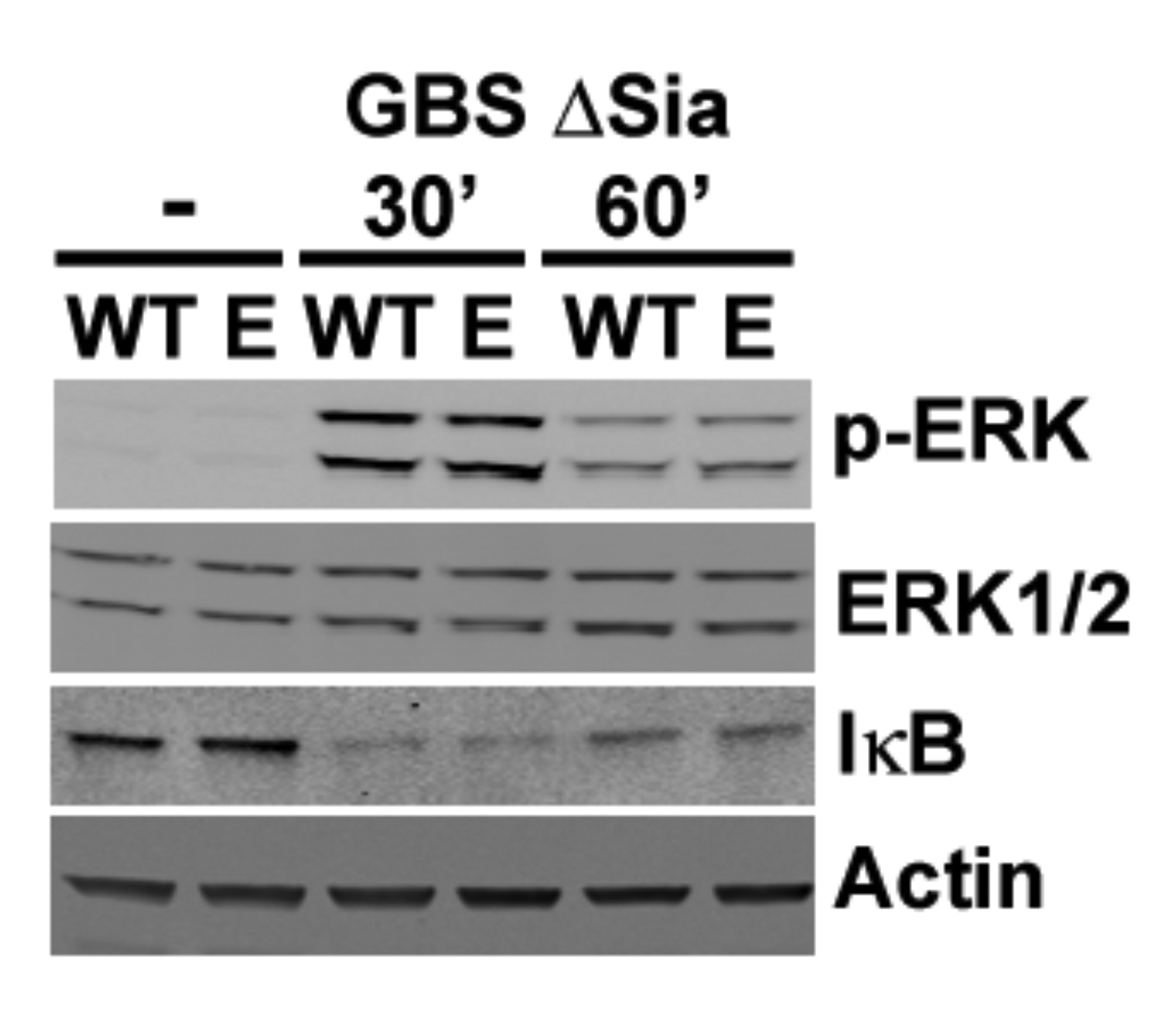

Supplement: Figure S4 — GBS ΔSia mutants induce similar level of ERK activation and IκB degradation in WT and Siglec-E deficient macrophages. WT mouse bone marrow-derived macrophages from WT (WT) or Siglec-E deficient (E) were treated with GBS ΔSia mutants for 30 or 60 min. Cell lysates were collected at indicated times, separated on SDS-PAGE, and probed with antibodies recognizing phosphorylated form of ERK, total ERK, IκB and actin. (TIF) [file ppat.1003846.s004.tif]

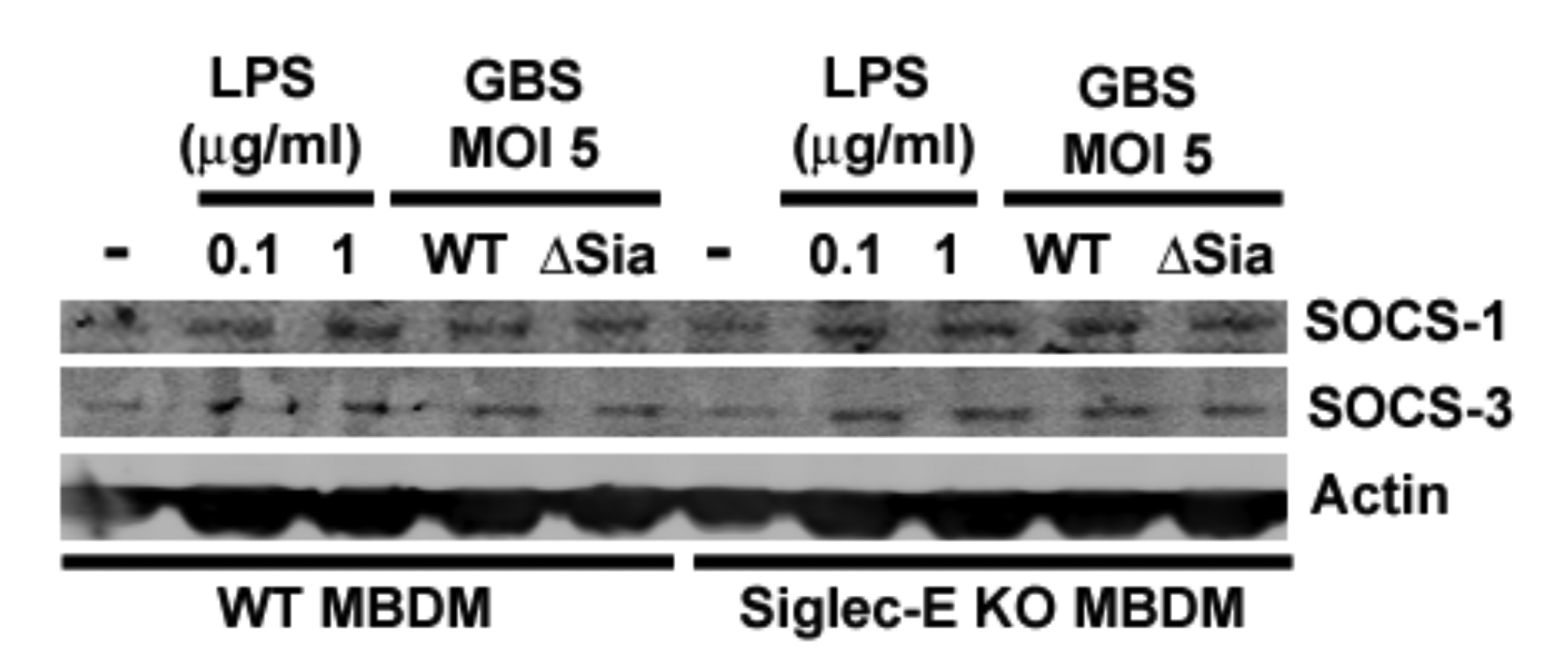

Supplement: Figure S5 — Expression of suppressor of cytokine signaling -1 (SOCS-1) and SOCS-3 in WT and Siglec-E deficient macrophages can be detected after LPS and GBS stimulation. Mouse bone marrow-derived macrophages (MBDMs) were stimulated with LPS, GBS WT and GBS ΔSia mutants for 24 h in the presence of antibiotics to prevent the overgrowth of bacteria. Cell lysates were collected, separated on SDS-PAGE, and probed with antibodies recognizing SOCS-1, SOCS-3 and actin. (TIF) [file ppat.1003846.s005.tif]

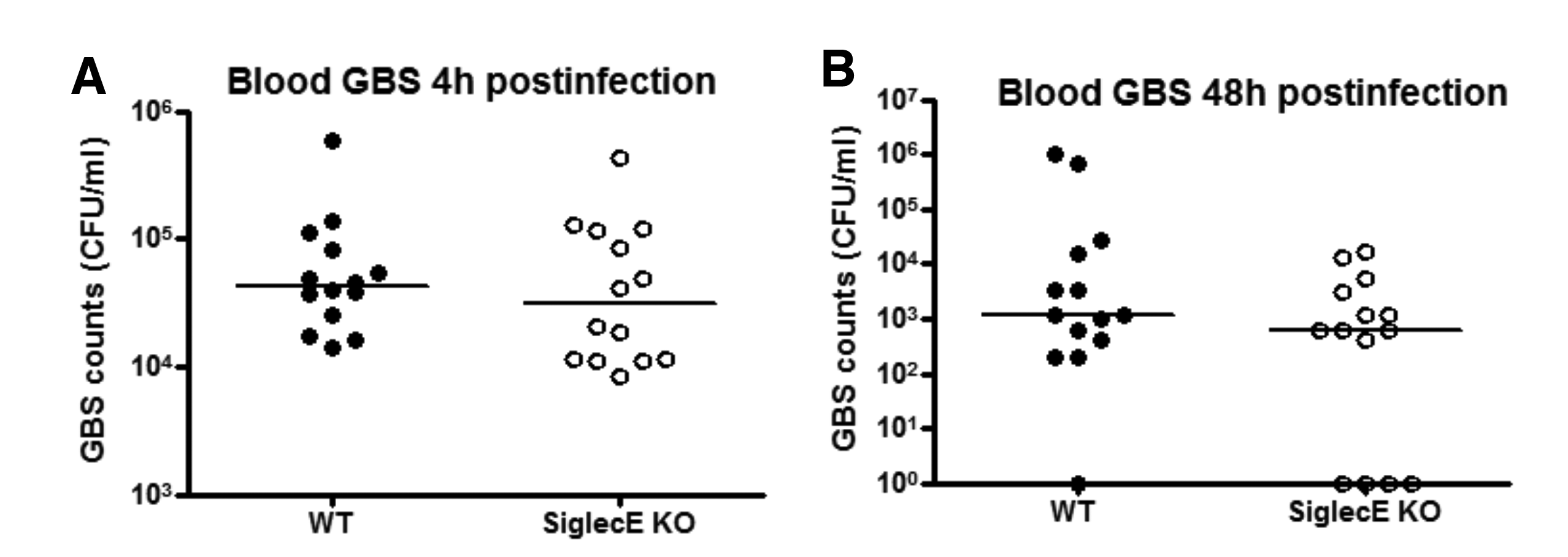

Supplement: Figure S6 — Similar blood GBS counts were observed in the systemic GBS infection model. Comparison of bacterial counts (expressed in CFU) recovered from the blood collected from WT and Siglec-E deficient mice after intravenous challenge with 108 CFU of WT GBS 4 h (A) or 48 h (B) after infection. Data shown are means and each circle denotes 1 mouse (n = 14 for each group). (TIF) [file ppat.1003846.s006.tif]

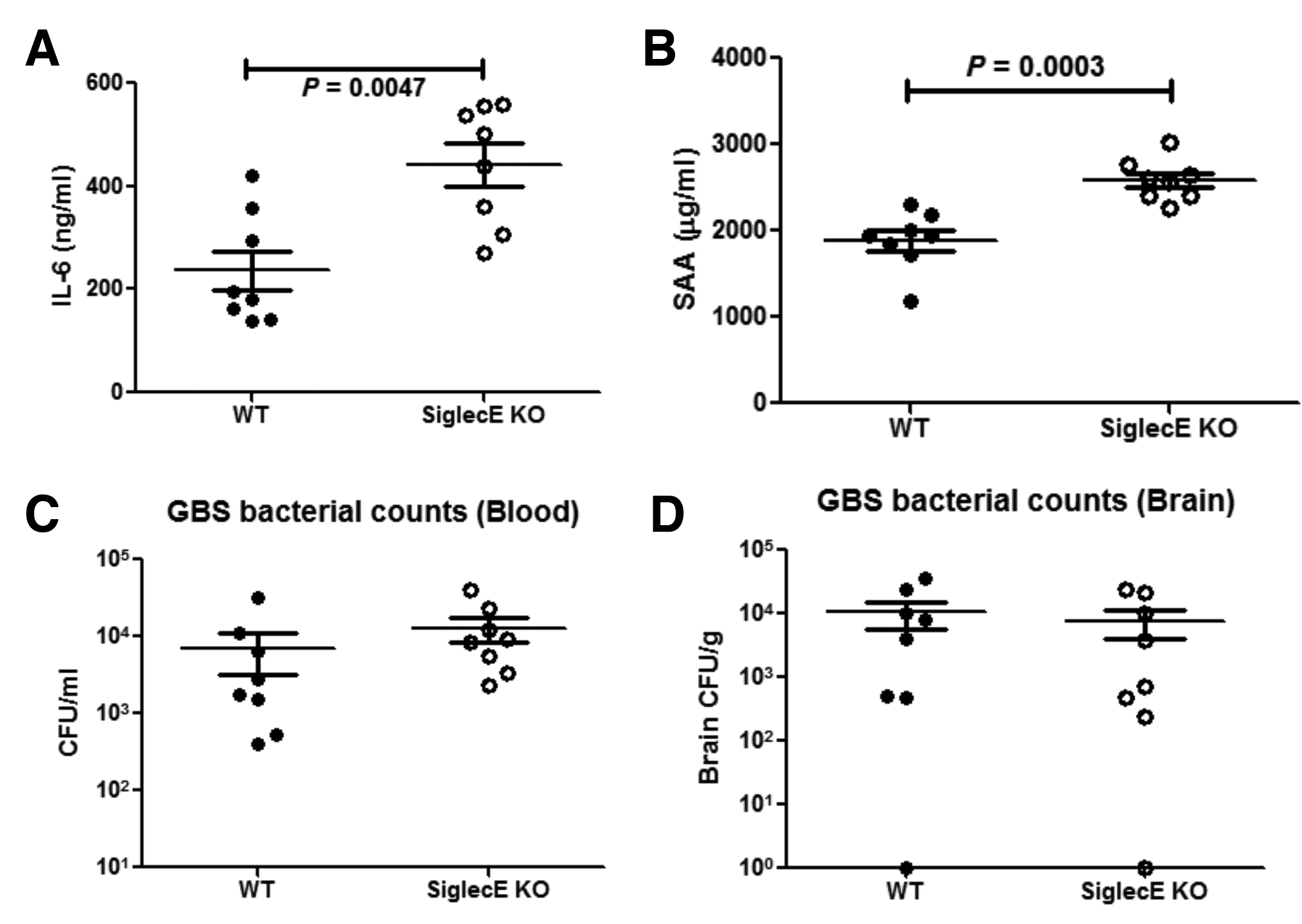

Supplement: Figure S7 — Enhanced proinflammatory cytokine secretion but equivalent bacterial brain dissemination in Siglec-E deficient mice upon sublethal GBS challenge at an early infection time point. WT and Siglec-E KO mice were challenged intravenously with 108 CFU of WT GBS. Blood and brains were collected 18 h after GBS infection to measure (A) serum IL-6 (A), (B) serum amyloid A (SAA), (C) blood and (D) brain bacteria loads. Data shown are means ± SEM and each circle denotes 1 mouse (n = 8 for each group). Differences between different groups were calculated by Mann-Whitney test. (TIF) [file ppat.1003846.s007.tif]
